# Stem cell transplantation for type 1 diabetes mellitus

**DOI:** 10.1186/1758-5996-1-4

**Published:** 2009-09-16

**Authors:** Júlio C Voltarelli, Carlos Eduardo Barra Couri

**Affiliations:** 1 Department of Clinical Medicine, Ribeirão Preto School of Medicine, University of São Paulo, Brazil

## Abstract

**Background:**

The use of stem cells to treat type 1 diabetes mellitus has been proposed for many years, both to downregulate the immune system and to provide β cell regeneration.

**Conclusion:**

High dose immunosuppression followed by autologous hematopoietic stem cell transplantation is able to induce complete remission (insulin independence) in most patients with early onset type 1 diabetes mellitus.

## Introduction

Type 1 diabetes (T1DM) is an autoimmune disease through which the patient's immune system loses the immunologic tolerance against β cells antigens, resulting in an immune response against the islets which involves CD8^+ ^and CD4^+ ^cells. Such process results in insulitis, an inflammatory process which causes the destruction of pancreatic β cells, thereby eliciting insulin therapy as the treatment for T1DM [1,2].

Stem cells (SC) have the unique function of asymmetric division, i.e., they are capable of self-renewing and the generation of other identical cells. They are undifferentiated cells that perpetuate throughout the body and are responsible for regenerating all tissues in adults and are also responsible for tissue morphogenesis in embryos. Such cells may be obtained from the fetus, the umbilical cord, the bone marrow and may also be deployed by peripheral blood and even from different somatic tissues[3,4].

Bone marrow SC originates blood cells, including lymphoid cells. In addition to hematopoietic stem cells, which are the most widely known and studied types of bone marrow SC, other important SC types have promising importance in therapeutics. One of them is the endothelial precursor cells, capable of angiogenesis and they are widely used nowadays in regenerative therapy. Another type of SC is the mesenchymal stem cell, which has a powerful immunosuppressant capacity and is also capable to differentiate into several tissues. Therefore, these three types of stem cells, hematopoietic, endothelial and mesenchymal, have been used in therapy and may be applied to the treatment of diabetes[5].

The bone marrow transplantion or classic stem cell transplantion has been performed successfully for exactly 40 years since the first successful transplant was performed in 1968. This type of transplantation is typically performed using SC mobilized from bone marrow cells to the peripheral blood or from the umbilical cord of an allogeneic donor, and transplanted to a patient who most of the times has hematologic disease. This type of transplant is preceded by immunosuppression in order to avoid rejection and the graft reaction against the host. Oppositely from solid organ transplants, the hematopoietic SC transplant requires limited continuous immunosuppression which may range from three to six months, depending on the pathology. In this type of transplant, a mutual tolerance is established between the donor and the recipient, due to chimerism, which characterizes donor cell persistence in the receptor. Chimerism results in a process which still remains unclear, associated with clonal exhaustion or clonal depression, which makes the receptor not to react to the donor or vice versa. Regarding the induction of chimerism in allogeneic transplantation, there are two types of transplant: myeloablative and nonmyeloablative. The first is the classic transplant, in which a very high dosage of debulking agents (chemotherapy and/or radiotherapy) is used to completely eliminate the hematopoietic and lymphoid tissues of the receptor. After that, these tissues are replaced by donor tissue, resulting in complete chimerism. Due to its intensity, myeloablative immunosuppression is highly toxic, resulting in non-eligible mortality rates. Currently, the nonmyeloablative transplant has been increasingly used, where the immunosuppression load is lower resulting in a mixed chimerism which is sufficient to avoid allogeneic reactions graft rejections against the host.

Such nonmyeloablative transplant strategy with partial chimerism induction is one hope to induce tolerance in the long run for solid organ transplants, such as kidney or pancreas transplants, but it is yet to be obtained. A paper written almost ten years ago, showed advantage while using bone marrow SC along with solid organ transplants in kidney-pancreas transplants in terms of chronic rejection, acute rejection and even corticoid interruption [6,7].

Recently in 2008, there were some reports of cases of tolerance induction including interruption of immunosuppresion after kidney or liver transplantation [8,9]. These reports showed the interruption of immunosuppresion in four out of five patients. In this nonmyeloablative conditioning procedure, a smaller dose of immunosuppressant drugs was used, which is not enough to completely eliminate recipient hematopoiesis. Such nonmyeloablative conditioning process is used to allow bone marrow grafting and to induce chimerism deployment. It gives us the hope that in the near future we may reach the goal of eliminating both rejection and graft reaction against hosts, resulting in the possibility of interrupting immunosuppression.

In the near future, according to these papers, allogeneic stem cell transplant has the perspective of allowing the grafting of other solid organs and the interruption of immunosuppression much like it is routinely performed in hematopoietic stem cell transplants as described in the literature [10].

### Autologous hematopoietic stem cell transplantation for type 1 diabetes

Another type of transplant which has been more widely used clinically, mainly in autoimmune diseases, such as T1DMs, is the autologous hematopoietic stem cell transplantion (AHSCT). For such transplant, we mobilize the patient's hematopoietic SC from bone marrow to the blood with the use of low dose cyclophosphamide and granulocyte colony-stimulating factor. Then the hematopoietic SC are collected from peripheral blood by leukapheresis and cryopreserved. The cells are re-injected intravenously only after conditioning with high dose of chemotherapy - cyclophosphamide (200 mg/kg) and rabbit antithymocyte globulin (4.5 mg/kg). This is a lymphoablative scheme, as we destroy most of the patient's lymphocyte clones, which include both autoreactive and non-autoreactive, and we recover the immunologic system with AHSCT. This phenomenon is called immunologic reset.

Such type of transplant has been performed for twelve years for autoimmune diseases such as multiple sclerosis, systemic sclerosis, rheumatoid arthritis, systemic lupus erythematous, Crohn's disease and others. This type of treatment has significant mortality rates, mainly for more severe diseases such as systemic sclerosis and lupus. In most cases, except for rheumatoid arthritis in adults, there is a prolonged remission in over 50% of the cases, which may be considered a success, as transplanted patients are the most severe cases and generally have not responded to usual therapies. In Brazil, a cooperative study protocol was initiated in 2001 and our series comprises is comprised of more than 150 patients, including patients with diabetes, multiple sclerosis, systemic sclerosis and lupus.

In animal models, T1DM may be prevented by allogeneic bone marrow transplant, and in allogeneic transplants in human beings, it has been shown that type 1 diabetes may be transmitted through bone marrow transplants. For example, a leukemic patient, after receiving a bone marrow transplant from a brother who has diabetes, may also become a diabetic patient, which means that the disease may be transmitted through stem cells. However, when type 1 diabetic patients received transplants due to leukemia or other blood-borne diseases, there was no improvement of diabetes after the transplant, which is contrary evidence to what has been observed in animal models. These patients have already had diabetes for several years, and they had small amount of β cell mass, demonstrating that the hematopoietic stem cell involved in such transplant is not able to differentiate into a significant number of β cells and to induce remission in the patient with long term disease.

The beneficial effect of several chronic immunosuppressant schemes was demonstrated in diabetes, however, after immunosupression interruption, the disease relapses [11-16]. Several studies tried to induce prolonged immunosuppression after a short term therapy with immunomodulatory drugs. One of the most successful results obtained with acute immunossuppression was with anti- T cell antibodies, as shown in articles reporting the effects of OKT3 in increasing C-peptide levels and a decreasing in daily insulin doses [17,18]. However such effect was transitory and too short.

As previously discussed, strategies of immunomodulation are more interesting during a phase in which the patient still has an important β cell reserve, i.e., in an initial stage of the disease, blocking the immunological aggression of T cells against β cells and allowing an endogenous regeneration of β cells. In face of this, our research group started a protocol of nonmyeloablative AHSCT in newly diagnosed type 1 diabetic patients in 2003 to transplant individuals from 12 to 35 years old with recent clinical and laboratorial diagnosis of type 1 diabetes (<6 weeks). In the initial protocol, especially in the first patient, we used corticoids along with rabbit antithymocyte globulin to avoid anaphylactic reactions. In face of the pro-apoptotic effects of glucocorticoids on β cells, we stopped use after that.

All the enrolled patients were checked for HLA hystocompatibility system antigens, and most of whom were DR3/DR4 [19].

## Side Effects and Results

Because we apply immunosuppressant leukemia therapy on an autoimmune disease in young patients, reports of side effects are extremely important, and the most frequent side effects are infections in general, controlled using an antibiotic scheme prophylatically or therapeutically. In three patients, we observed post-transplant autoimmune diseases due to the transplant or to diabetes.

During a 7- to 58-month follow-up (mean, 29.8 months; median, 30 months), 20 patients without previous ketoacidosis and not receiving corticosteroids during the preparative regimen became insulin free. Twelve patients maintained this status for a mean 31 months (range, 14-52 months) and 8 patients relapsed and resumed insulin use at low dose (0.1-0.3 IU/kg). In the continuous insulin-independent group, A1c levels were less than 7.0% and mean (SE) area under the curve (AUC) of C-peptide levels increased significantly from 225.0 (75.2) ng/mL per 2 hours pretransplantation to 785.4 (90.3) ng/mL per 2 hours at 24 months posttransplantation (*P *< .001) and to 728.1 (144.4) ng/mL per 2 hours at 36 months (*P *= .001). In the transient insulin-independent group, mean (SE) AUC of C-peptide levels also increased from 148.9 (75.2) ng/mL per 2 hours pretransplantation to 546.8 (96.9) ng/mL per 2 hours at 36 months (*P *= .001), which was sustained at 48 months. In this group, 2 patients regained insulin independence after treatment with sitagliptin, which is a DPP-4 inhibitor, an enzyme which metabolizes GLP-1, and is thus associated with a glucose dependent increase in C-peptide levels. Two patients developed bilateral nosocomial pneumonia, 3 patients developed late endocrine dysfunction, and 9 patients developed oligospermia. There was no mortality [19,20].

## Conclusion

We have concluded that there are several challenges when it comes to this type of transplant in recently diagnosed T1DM, from which the main one is to investigate the length of the clinical response (insulin independence) and relapse mechanism. Using embryonic stem cells could have better results at the long term diseases. Injecting bone marrow mononuclear cells directly into the pancreas significantly increased endogenous insulin secretion in type 2 diabetic patients, but not in persons with T1DM in studies conducted in Peru and Argentina. The use of autologous umbilical cord stem cells in children with T1DM resulted in not significant differences in daily insulin doses and in a decline in C-peptide levels after 1 year of follow-up. This study is still being conducted at the University of Florida, in Gainesville.

## Competing interests

The authors declare that they have no competing interests.

## Authors' contributions

All the authors and participated in the manuscript design and drafting. All authors participated in study coordination and acquisition and interpretation of the data showed. All authors read and approved the final manuscript.
